# The Ratio of the Size of the Abdominal Aortic Aneurysm to That of the Unchanged Aorta as a Risk Factor for Its Rupture

**DOI:** 10.3390/biomedicines10081997

**Published:** 2022-08-17

**Authors:** Maciej Jusko, Piotr Kasprzak, Alicja Majos, Waclaw Kuczmik

**Affiliations:** 1 Department of General Surgery, Vascular Surgery, Angiology and Phlebology, Medical University of Silesia, 40-055 Katowice, Poland; 2 Department of Vascular Surgery, University Hospital Regensburg, 93053 Regensburg, Germany; 3 General and Transplant Surgery Department, Medical University of Lodz, 93-338 Lodz, Poland

**Keywords:** ruptured abdominal aortic aneurysm, aneurysm rupture risk factors, aneurysm rupture risk assessment

## Abstract

Background: A ruptured abdominal aortic aneurysm is a severe condition associated with high mortality. Currently, the most important criterion used to estimate the risk of its rupture is the size of the aneurysm, but due to patients’ anatomical variability, many aneurysms have a high risk of rupture with a small aneurysm size. We asked ourselves whether individual differences in anatomy could be taken into account when assessing the risk of rupture. Methods: Based on the CT scan image, aneurysm and normal aorta diameters were collected from 186 individuals and compared in patients with ruptured and unruptured aneurysms. To take into account anatomical differences between patients, diameter ratios were calculated by dividing the aneurysm diameter by the diameter of the normal aorta at various heights, and then further comparisons were made. Results: It was found that the calculated ratios differ between patients with ruptured and unruptured aneurysms. This observation is also present in patients with small aneurysms, with its maximal size below the level that indicates the need for surgical treatment. For small aneurysms, the ratios help us to estimate the risk of rupture better than the maximum sac size (AUC: 0.783 vs. 0.650). Conclusions: The calculated ratios appear to be a valuable feature to indicate which of the small aneurysms have a high risk of rupture. The obtained results suggest the need for further confirmation of their usefulness in subsequent groups of patients.

## 1. Introduction

A ruptured abdominal aortic aneurysm (RAAA) is a major challenge in vascular surgery. Abdominal aortic aneurysm (AAA) itself is usually asymptomatic until the moment of its rupture, when the patient’s circulatory capacity suddenly collapses due to massive internal hemorrhage, and even despite the use of proper surgical treatment, serious health complications may often develop, and mortality is between 32–70% [1]. Due to such a high mortality, the effort of research teams should be focused on the most accurate estimation of which patients with AAA are most commonly predisposed to the development of aneurysm rupture and when, and therefore when they should undergo treatment in order to avoid this most serious complication [2,3,4]. Unfortunately, despite many years of experience in AAA treatment and numerous trials, it is difficult to predict when the aneurysm will rupture [5,6,7,8].

The rupture of the aneurysm wall is a complex process, influenced by many factors with complex relations, which to a large extent makes it difficult to fully explain the problem [7]. In recent years, in the light of new achievements, the concept of the RAAA development mechanism has changed several times. New discoveries allowed us to answer many yet-unanswered questions, but they led to many more, often much more complex ones [9,10,11,12,13]. Some of the considerations regarding the mechanisms by which aneurysm rupture occur are presented below.

It was originally assumed that a key role in this phenomenon was played by the pressure gradient on both sides of the vascular wall, which the aneurysm was unable to balance. The pressure exerted by the blood flowing through the aorta on its wall was calculated according to Laplace’s law, which explains the way the liquid flows through cylindrical objects. Although this law describes the conditions in the physiological aorta relatively well, due to the extensive remodeling of the vessel, which unevenly affects different areas of the aorta, it cannot be used to describe the conditions in an aneurysm [14]. This prompted researchers to look for a method that could determine the local tensions of the AAA wall taking into account its often complicated shape. This was achieved thanks to the finite element method (FEM), which allowed the identification of the most vulnerable areas of the aortic wall (the so-called hot spots). FEM made it possible to better estimate which AAAs are more likely to rupture compared to Laplace’s law, but in this technique the analysis is based primarily on the shape of the vessel itself. In the course of further research, it turned out that the local wall tension largely depends not only on the AAA shape and its extension, but also on the remodeling of the vascular wall [15,16]. Inside the AAA wall, a complicated inflammatory process takes place, the consequence of which is the reduction in its strength, which, to a large extent, may be the cause of its rupture. The rebuilding of the AAA wall mainly consists of smooth muscle cell atrophy, degradation of elastin fibers, increased collagen synthesis and increased neovascularization [12,13].

An additional difficulty in estimating the risk of RAAA development is the presence and size of intraluminal thrombus (ILT). According to some reports, this is a factor preventing the aneurysm from rupture, as it is a layer that isolates the AAA wall from the flowing blood [17,18]. There are also publications reporting an increased risk of RAAA development in the presence of ILT, as ILT as a condensed object is a good conductor for the stresses on the AAA wall [19]. Moreover, it has been found that the cells of the internal vascular wall are to a certain extent supplied with oxygen and nutrients by the blood flowing inside the vessel independently of the vasa vasorum. In such a case, ILT, by blocking the access of blood to the endothelium, determines the formation of an ischemic zone, which may intensify the inflammatory reaction and contribute to the weakening of aneurysm wall strength [20,21,22].

The combination of all the above-mentioned factors and the estimation of the risk of rupture on their basis is possible and provides valuable data; however, it causes many difficulties in assessment and is hard to perform. The reports so far that take into account most of the factors of RAAA development, i.e., aneurysm morphology, the presence of ILT and the histological structure of the vessel wall, were retrospective, and therefore cognitive. A diagnostic protocol based on the above data allowing us to modify the current indications for AAA treatment has not been developed so far and the maximum diameter of the aneurysm remains the main factor determining whether a certain patient will be treated or not [23].

The correlation between AAA diameter and the risk of its rupture is a well-known observation [5,6]. In order to measure the maximum AAA diameter, it is sufficient to analyze a single CT scan of the abdominal aorta and to perform a single measurement. The ease of making this estimation means that almost all recommendations and guidelines are mainly based on these data [23,24]. However, estimating this risk based on the diameter alone is burdened with an error resulting from not considering other factors. The same CT scan, after a more detailed analysis, may provide additional valuable data that can be used to estimate the risk of AAA rupture. Kimura et al. were comparing CT scans of patients with AAA and RAAA, and they found that the ruptured aneurysms are characterized by a smaller rounding radius (departure angle) and a smaller aspect ratio (longitudinal diameter divided by transverse) [25]. Such observations lead us to a deepening of the analysis of data that can be obtained from a CT scan, because finding the characteristic features of RAAA other than the maximum diameter could increase the number of patients qualifying for treatment.

The initial point of this study was to question the need of sticking to the strict limits of maximum AAA diameter as the main indication for surgical treatment, which does not take into account anatomical variability between individual patients other than gender difference. Evidence of the limitations of that approach is the relatively high percentage of RAAA in patients with non-qualifying diameters identified in published studies [8,26,27]. With the current availability of healthcare services, it should be expected that some of these patients had been diagnosed with AAA and were not qualified for surgical treatment prior to aneurysm rupture.

One of the reasons for this may be the presence of different diameters of the aorta in patients with the same size of AAA. When defining an aneurysm, we base on the ratio of the diameter of a healthy artery to the dilated segment. The hypothesis of this research is potential greater risk of rupture of AAA in the case of a smaller aorta than in the case of a larger one with aneurysms of the same size. For this reason, this study analyzes the diameters of not only AAA, but also the aorta and iliac arteries at different heights. We assume that the dimensions of the unchanged vessels closest to the aneurysm will allow us to take into account individual morphological differences, and their comparison will allow for a better assessment of the risk of aneurysm rupture.

It has not been fully determined if or how profoundly the presence or absence of AAA neck segment should be regarded. The term abdominal aortic aneurysm is defined as any significantly large dilation of the aorta, from the aortic hiatus to the division into the iliac arteries. The majority of these cases are patients with an aneurysm located below the exit of the renal arteries, as in this region the histological structure determines the greater susceptibility of the vascular wall to remodeling into an aneurysm [5,10]. A rarer variant is an aneurysm present in the segment of major visceral arteries departure, and such aneurysms are often treated in clinical practice as a separate disease, not because of a different etiopathogenesis, but a different method of their treatment. However, relatively often there is an indirect variant, i.e., aneurysm, which does not cover the section where the visceral arteries depart from the aorta but begins so close to the renal arteries that there is no undilated section between them and the aneurysm sac, i.e., the neck. In this article, such aneurysms are referred to as non-neck aneurysms. Due to the same localization of aneurysms with and without the neck, in the literature referred to as infrarenal, there are no reasons to exclude non-neck aneurysms from the study group. However, all results and conclusions obtained on the basis of the measurements of the aneurysm neck section should be treated with adequate reserve, as it has not been precisely established whether and when the neck is a physiological section of the vessel or if it is already a primary lesion.

## 2. Materials and Methods

The study group consisted of 93 patients with infrarenal RAAA, and the control group consisted of 93 patients with infrarenal AAA operated electively on the basis of the existing criteria. The diagnoses were confirmed by angio-CT scan of the abdominal aorta. All subjects were hospitalized in the Department of General Surgery, Vascular Surgery, Angiology and Phlebology of the Upper Silesian Medical Center, Medical University of Silesia in Katowice, Poland, in 2014–2019. The control group consisted of patients treated regularly due to AAA, successively in 2018–2019.

**Figure 1:**
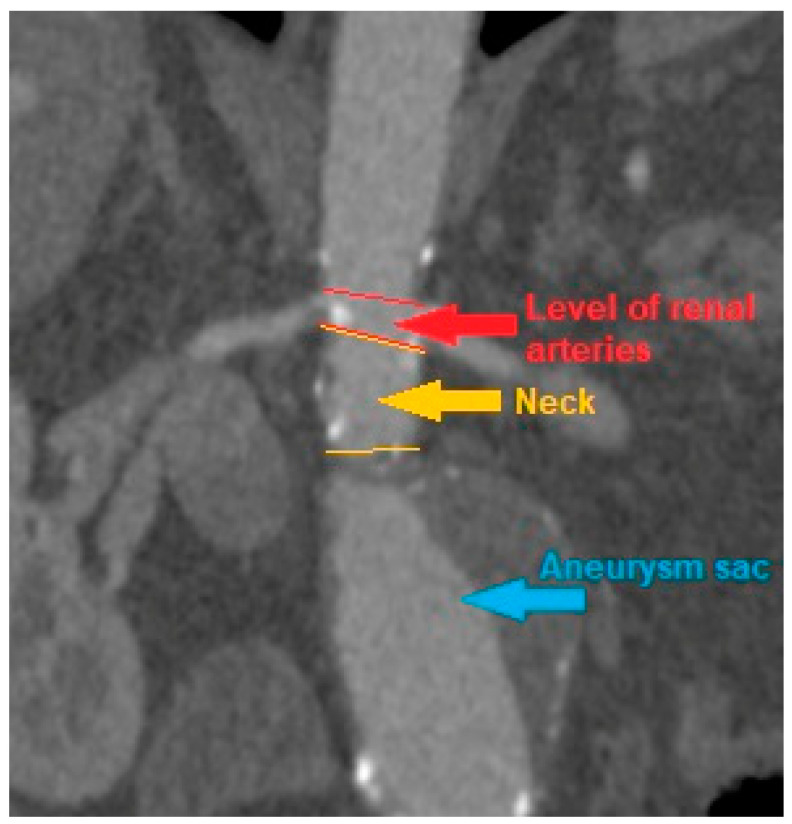
An example of computed tomography of patient with infrarenal abdominal aortic aneurysm with present neck segment.

CT scans were analyzed, and the AAA, iliac arteries and aortic diameter were measured at following levels: celiac trunk, superior mesenteric artery, renal arteries, the maximum diameter of the aneurysm sac, understood as the diameter of the AAA at the level with the highest sac circumference, the diameter of both common iliac arteries and both external iliac arteries. In addition, measurements were made of the AAA neck diameter, i.e., the diameter of the aortic section inclined at a right angle to its long axis at the level of the segment above the AAA sac and below the level of the renal arteries, provided that such segment was present at all due to AAA morphology. The diameter was measured between the outer edges of the vessel wall opposite to its center. If ILT was present inside an aneurysm sac it was also included in the measurement of maximal aneurysm diameter. The measurement was made with OsiriX DICOM Viewer program (Figure 1).

The measurement of the diameter of a ruptured abdominal aortic aneurysm and its reliability remains a problematic issue, since the original diameter of the aneurysm may have changed after the rupture. For obvious clinical reasons, it is not possible to observe how the diameter of the aorta changes during an aneurysm rupture and in time after this incident, and the only test that can be referred to in order to measure a ruptured aneurysm is a CT scan performed to diagnose this incident. The possible difference in the diameter of the aneurysm just before and after rupture and its influence on the results obtained is a potential limitation of the present study.

After measuring the diameters, the aortic diameter ratios at certain levels were calculated. The ratios are the quotient of the maximum diameter of AAA and the diameter of the aorta at the level of the celiac trunk, the superior mesenteric artery, the renal arteries, the diameter of both common iliac arteries, and the diameter of both external iliac arteries. The ratios were named as the “R” and the name of the artery at the level of which the aortic diameter was measured, or the common and external iliac artery with the additional definition of the side.

In order to reveal potential differences in calculated ratios, the subjects were divided into three groups according to the maximum diameter of the aneurysm. The first group consisted of patients with AAA diameter below 5 cm, which was the size below indications for surgical intervention according to current criteria. The second was patients with a maximum diameter of AAA in the range of 5–6.5 cm, and the third was patients with AAA larger than 6.5 cm. Due to the gender disproportion in the study group (18 vs. 168), expressed even stronger after the division into subgroups, and therefore the high probability of low reliability of the results obtained in the small group, division into women and men was not used for further calculations. Patients with AAA smaller than 4 cm were not included in the analysis, because in the study group all such aneurysms had a sac-like morphology. In such a case, when AAA is not a dilation of the entire circumference of the aortic wall, but only a short segment bulge of a certain part, it seems that its rupture may be determined by other factors or of a different intensity than in the analyzed, fusiform aneurysms. To keep consistency, the study did not concern any sac-like aneurysms, regardless of their size. Additionally, subjects with iliac artery aneurysm understood as widening of the common iliac artery over 2 cm were not included in the study.

The study group and the control group were compared in terms of differences in the AAA measurement values and the calculated ratios. Next, the predictive value for the aneurysm rupture event of calculated ratios and the maximum diameter of the aneurysm was compared. The distribution of the values of the ratios in relation to the amount of RAAA subjects, which occur at a given ratio value, was analyzed. Due to the fact that the aneurysm neck segment was present only in some of the subjects, the above comparisons were made separately for the subgroups of subjects with and without the neck segment.

When comparing continuous variables, the normality was determined with the Shapiro–Wilk test. For the data that met the assumptions of the normal distribution, the groups were compared with the Student’s t-test, for the data that did not meet the assumptions of the normal distribution, the Mann–Whitney U test was used. The quoted effect measures were calculated using ROC curves. The calculations were made using the Statistica 13 program.

## 3. Results

The group consisted of 18 women and 168 men. In the study group, the minimum diameter of RAAA was 4.4 cm, the maximum was 14.2 cm, and the median was 7.4 cm. In the control group the minimum diameter of AAA was 4.2 cm, the maximum was 9.5 cm, and the median was 5.6 cm. A total of 37 patients did not have a segment of the aorta that could be referred as an aneurysm neck; therefore, for the calculation with the use of ratio neck, the group was reduced to 149 subjects.

**Figure 2:**
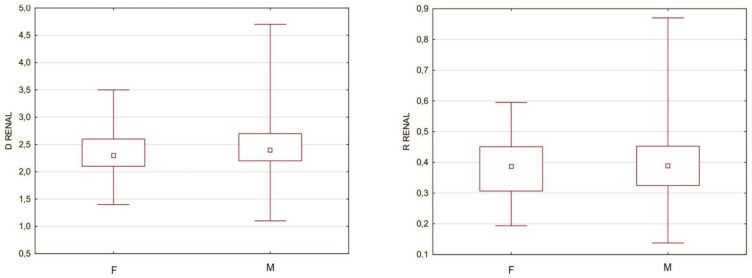
A comparison of aorta diameter (D RENAL) and renal ratio (R RENAL) between women (F) and men (M). D Renal for women from 1.4 to 3.5 cm with majority from 2.1 to 2.6 cm, D Renal for men from 1.1 to 4.7 cm with majority from 2.2 to 2.7 cm. R Renal for women from 0.2 to 0.6 with majority from 0.3 to 0.45, R renal for men from 0.14 to 0.88 with majority form 0.32 to 0.45. All differences insignificant *p* > 0.05.

A problem that would not allow for all subjects to be gathered into a single cohort were the potential differences in aortic structure and aneurysm morphology between men and women, which are included in the current guidelines for the treatment of abdominal aortic aneurysms. To determine the validity of such a fusion, among groups of men and women, the separate sex subgroups were extracted, which were characterized by the same minimum and maximum diameter of the aneurysm. There were 17 women and 162 men with a minimum aneurysm diameter of 4.4 cm and a maximum diameter of 9.8 cm. The mean size was 6.4 cm for women and 6.5 cm for men, and the median was 5.8 cm for women and 6.1 cm for men. The aorta diameter above the aneurysm was compared between men and women, and for this purpose, the diameter of the aorta at the level of renal arteries was arbitrarily used for some subjects that did not have the neck segment. To simultaneously take into account the size of the aneurysm, the R Renal was compared as well. The analysis revealed no statistically significant differences both when comparing the diameter and the ratio; therefore, further measurements were made without gender division (Figure 2).

**Figure 3:**
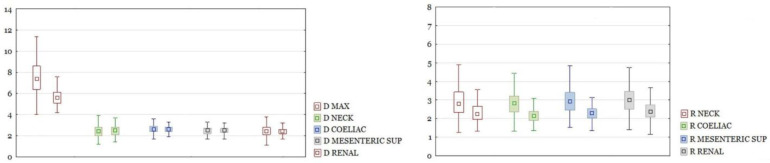
On the left, a comparison of diameters of the aorta at different levels and AAA diameters between ruptured and unruptured aneurysms in the whole cohort. Diameters in cm. Boxplots are shown in pairs. The left one is diameter for ruptured, the right one is diameter for unruptured aneurysms. Only D MAX difference is significant with *p* < 0.05. On the right, a comparison of ratios between ruptured and unruptured aneurysms. Boxplots are shown in pairs. The left one is ratio for ruptured, the right one is ratio for unruptured aneurysms. All differences significant at *p* < 0.05.

**Figure 4:**
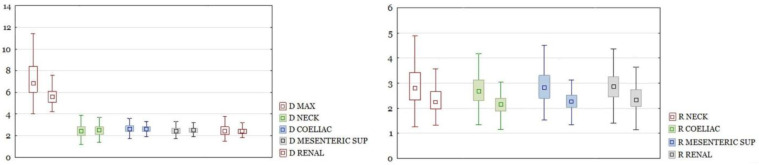
On the left, a comparison of diameters of the aorta between ruptured and unruptured aneurysms with the neck segment present. Diameters in cm. Boxplots are shown in pairs. The left one is diameter for ruptured, the right one is diameter for unruptured aneurysms. Only D MAX difference is significant with *p* < 0.05. On the right, a comparison of ratios between ruptured and unruptured aneurysms. Boxplots are shown in pairs. The left one is ratio for ruptured, the right one is ratio for unruptured aneurysms. All differences significant at *p* < 0.05.

**Figure 5:**
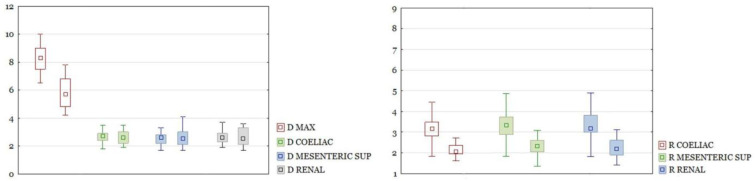
On the left, a comparison of diameters of the aorta between ruptured and unruptured aneurysms with the neck segment absent. Diameters in cm. Boxplots are shown in pairs. The left one is diameter for ruptured, the right one is diameter for unruptured aneurysms. Only D MAX difference is significant with *p* < 0.05. On the right, a comparison of ratios between ruptured and unruptured aneurysms. Boxplots are shown in pairs. The left one is ratio for ruptured, the right one is ratio for unruptured aneurysms. All differences significant at *p* < 0.05.

RAAA in the entire study group had a larger maximal diameter than AAA in the control group. The differences in other section sizes were not significant. The differences of all calculated ratios between the study group and the control group were statistically significant (Figure 3). There were the same results for those parameters after dividing the group into neck and non-neck subgroups (Figure 4 and Figure 5).

**Figure 6:**
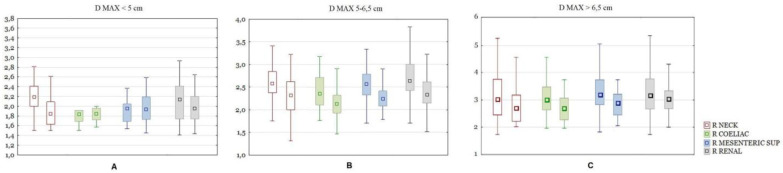
(**A**) A comparison of ratios of the aorta between ruptured and unruptured aneurysms with the neck segment present. Subgroup with D MAX < 5 cm. Boxplots are shown in pairs. The left one is ratio for ruptured, the right one is ratio for unruptured aneurysms. Only the R NECK difference is significant with *p* < 0.05. (**B**) A comparison of ratios of the aorta between ruptured and unruptured aneurysms with the neck segment present. Subgroup with D MAX 5–6.5 cm. Boxplots are shown in pairs. The left one is ratio for ruptured, the right one is ratio for unruptured aneurysms. All differences significant at *p* < 0.05. (**C**) A comparison of ratios of the aorta between ruptured and unruptured aneurysms with the neck segment present. Subgroup with D MAX > 6.5 cm. Boxplots are shown in pairs. The left one is ratio for ruptured, the right one is ratio for unruptured aneurysms. Only the R NECK difference is significant with *p* < 0.05.

As mentioned above, the test and control groups were divided into size ranges (<5; 5–6.5; >6.5 cm) and the previous calculations were made for each subgroup separately. In the group of subjects with the present aneurysm neck segment, the maximum size of the aneurysm differed significantly between the test and control groups in the size ranges of the aneurysm sac <5 cm and 5–6.5 cm. When comparing the difference in the ratios, for aneurysms smaller than 5 cm in diameter, significant differences were shown only for Ratio Neck. In the size range from 5–6.5 cm, the following ratios were significantly different: Ratio Celiac, Mesenteric, Renal, Neck, Left Iliac comm and Left iliac ext, with the difference of the latter two being less pronounced. For aneurysms larger than 6.5 cm in diameter, significant differences were shown only for the Ratio Neck (Figure 6).

In the group of subjects without the aneurysm neck segment, after dividing the group into size subgroups, there were too few subjects in each one of them for the results to be relied upon.

**Figure 7:**
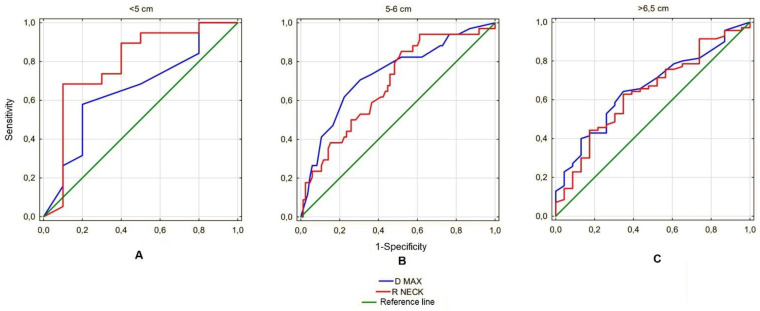
(**A**) ROC curves for the predictive value of R NECK compared with D MAX and the reference line in the group of aneurysms with D MAX < 5 cm. AUC for D MAX: 0.65, AUC for R NECK: 0.783. (**B**) ROC curves for the predictive value of R NECK compared with D MAX and the reference line in the group of aneurysms with D MAX 5–6.5 cm. AUC for D MAX: 0.729, AUC for R NECK: 0.68. (**C**) ROC curves for the predictive value of R NECK compared with D MAX and the reference line in the group of aneurysms with D MAX > 5 cm. AUC for D MAX: 0.658, AUC for R NECK: 0.641.

Next, the analyzed ratios were assessed for predictive value for AAA rupture. Since the only ratio showing significant differences in each of the size subgroups was the Ratio Neck, the predictive value was calculated for this parameter only and compared with the prognostic value of the maximum aneurysm sac diameter, the parameter currently used to estimate the risk of rupture. This analysis revealed that in the <5 cm subgroup, the Ratio Neck had a better prognostic value for predicting the risk of rupture than the maximum sac diameter (AUC 0.783 vs. 0.650). In the range 5–6.5 cm, the maximum diameter was a better predictor for estimating risk of rupture than the Ratio Neck (AUC 0.680 vs. 0.729), and in the >6.5 cm subgroup both parameters showed similar prognostic value for this phenomenon (AUC 0.641 vs. 0.658) (Figure 7).

**Figure 8:**
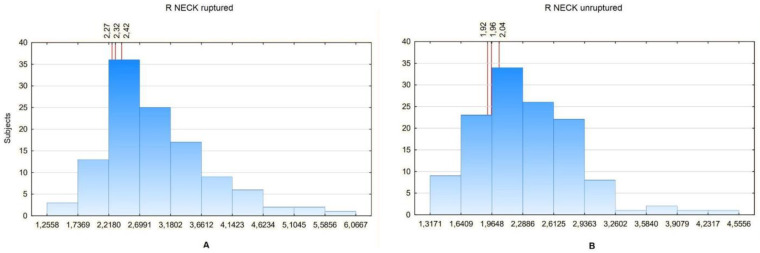
(**A**) R NECK values distribution in a certain number of patients with ruptured aneurysm. For the 20th percentile: 2.27, 25th percentile: 2.32, 30th percentile: 2.42. (**B**) R NECK values distribution in a certain number of patients with unruptured aneurysm. For the 20th percentile: 1.92, 25th percentile: 1.96, 30th percentile: 2.04.

Then, the values of particular ratios in a certain percentage of patients were analyzed. To compare them between the groups, the ratio values in the 20th, 25th and 30th percentiles were analyzed. The Ratio Neck was used for the analysis, as it revealed significant differences between the study and control group in all aneurysm size subgroups. Since the Ratio Neck can only be calculated for aneurysms with a neck segment, the Ratio Renal values were additionally compared, as it would seem that it most closely describes the relation between an aneurysm sac and unchanged aorta in the patients without a neck segment. The comparison showed that in general the ratios tend to be lower for non-ruptured aneurysms. The Ratio Neck was 2.26 for 20%, 2.3 for 25% and 2.4 for 30% for ruptured aneurysms and 1.9 for 20%, 1.94 for 25% and 2.01 for 30% for non-ruptured aneurysms (Figure 8).

**Figure 9:**
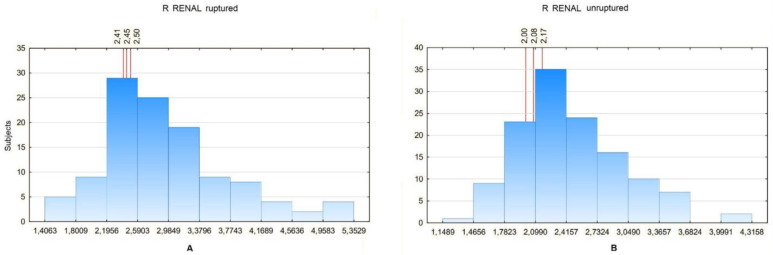
(**A**) R RENAL values distribution in a certain number of patients with ruptured aneurysm with neck segment present. For the 20th percentile: 2.41, 25th percentile: 2.45, 30th percentile: 2.5. (**B**) R RENAL values distribution in a certain number of patients with unruptured aneurysm with neck segment present. For the 20th percentile: 2, 25th percentile: 2.08, 30th percentile: 2.17.

The Ratio Renal analysis was performed separately for the subjects with and without the neck segment. For aneurysms with the neck segment present, the values were 2.4 for 20%, 2.41 for 25% and 2.49 for 30% for ruptured aneurysms and 2 for 20%, 2.05 for 25% and 2.18 for 30% for non-ruptured aneurysms (Figure 9).

**Figure 10:**
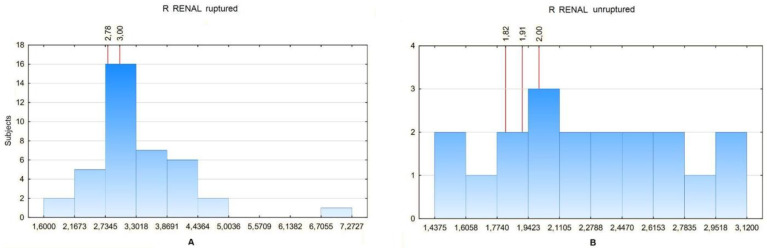
(**A**) R RENAL values distribution in a certain number of patients with ruptured aneurysm with neck segment absent. For the 20th percentile: 2.78, 25th percentile: 3, 30th percentile: 3. (**B**) R RENAL values distribution in a certain number of patients with unruptured aneurysm with neck segment absent. For the 20th percentile: 1.82, 25th percentile: 1.91, 30th percentile: 2.

For aneurysms without necks, the values were 2.78 for 20%, 3 for 25% and 3 for 30% for ruptured aneurysms and 1.8 for 20%, 1.91 for 25% and 2 for 30% for non-ruptured aneurysms (Figure 10).

## 4. Discussion

AAA is a pathological lesion with a very heterogeneous structure and shape when compared between individual patients. The differences in AAA morphology, the length and characteristics of the aortic section it covers, as well as the histological structure of the vascular wall make it reasonable to look for individual factors that would allow the patient to be provided with surgical treatment. There may be factors other than the maximum size of the aneurysm, which, according to the variables mentioned above, could in some cases lower the diameter importance [7,10,25]. In the current guidelines, the only manifestations of taking into account the individual characteristics of a certain patient are lowering the AAA size range due to the female gender and qualifying patients for surgical treatment with AAA, which increases its maximum size relatively quickly [23,24]. The latter factor, although important in clinical practice, turns out to be difficult to assess, as it requires at least two computed tomography (CT) scans per year in a specific patient. Given the widespread use of ultrasound diagnostics used in vascular outpatient clinics, providing a fairly good, but incomplete, picture of the disease, this is rarely practiced. Ultrasound measurements are subject to some error from the lack of standardization of this method, which may result in the delivery of many false positive and false negative results of a rapid increase in the maximum diameter of AAA [28]. Moreover, intravenous administration of a contrast agent, which, with frequent examinations, may cause or lead to an exacerbation of the present renal failure is not without significance [29]. Due to the above-mentioned difficulties, a group of patients may be mistakenly classified as low-risk RAAA patients, and they are not qualified for surgical treatment as they should be.

The starting point of this study is the assumption that even a single CT scan can provide more data indicating an increased risk of AAA rupture in a certain patient than the maximal aneurysm size alone. A study by Kimura et al. took into account, for example, the fillet radius and the aspect ratio, the parameters whose values, obtained through a single CT scan, distinguished RAAA from AAA [25]. In our study, emphasis was placed on taking into account the size of the unchanged aorta and iliac arteries and comparing them to the size of aneurysm sac. It was assumed that even in the case of a relatively small aneurysm, when there is also a small size of healthy vessels, the peak wall stress (PWS) may be significant. The normal abdominal aorta has a very similar diameter throughout its entire length down to the division into the iliac arteries. However, abdominal aneurysms commonly have diverse morphologies and may be accompanied by dilations in other parts of the aorta that are not sufficiently large to be classified as aneurysms. We therefore determined the dimensions of the aorta at several separate sites. Similarly, the calculations concerning the diameter of iliac arteries were enrolled.

The study assumes that the degree of enlargement of the aneurysm sac in relation to the initial size of the aorta is a significant risk factor for AAA rupture. Unfortunately, verifying the validity of this statement in each RAAA patient turns out to be difficult to enroll. The mean diameter of ruptured abdominal aneurysms encountered in clinical practice is larger than the threshold at which an aneurysm becomes eligible for surgical treatment Since no one undermines the benefits of surgical treatment in patients with large AAA, aneurysms of a size on the threshold of current indications for surgery and smaller have become the object of special interest in this study. Because of that, we divided our group into the subgroups based on the aneurysm size. Thanks to this division, it was possible to visualize the differences between patients with similar aneurysms but different aorta sizes, which was reflected in the calculated ratios. If no differences in the values of the ratios were observed, this would mean that the comparison of the size of the unchanged aorta to the size of the aneurysm is irrelevant and the only anatomical factor determining the rupture is the maximum size of the aneurysm sac. An interesting observation is the presence of numerous significant differences in the 5–6.5 cm subgroup, with only single ones in the remaining subgroups. In large aneurysms (>6.5) this may be due to the variety of sizes of the aneurysm sacs, still present in this subgroup even after division. This diversity does not allow us to prove the potential value of the ratios; however, in these subjects the mere fact of the presence of a large aneurysm indicates a risk of rupture high enough for the patient to be qualified for surgical treatment. The lack of differences in the values of the ratios in the group of small aneurysms (<5 cm) with only single exception indicates that there are no particular anatomical differences between ruptured and unruptured aneurysms of such size, so their rupture is rather induced by non-anatomical risk factors.

Another interesting observation is the comparison of the predictive value of the calculated ratios and the maximum AAA size for the estimation of the risk of rupture. It is noteworthy that in the studied group, in the size range <5 cm, the predictive value of maximum diameter of the aneurysm sac is characterized by the AUC for the ROC curve at the level of 0.655, which means that it has a slightly better predictive value for predicting AAA rupture than a coin toss (0.500) and does not meet the significance for a good aneurysm rupture risk predictor. The remaining parameter, R Neck, fares better in this respect with AUC 0.787, which in our opinion proves the predictive value is high enough to analyze this parameter in other study groups, as it could potentially become a new indication for surgical treatment of small aneurysms. In the remaining analyzed subgroups, the Ratio Neck size presented a prognostic value similar to or worse than the maximum AAA sac size, which limits its potential use.

In order to complete this analysis, it is necessary to look at what values each ratio has and what these values indicate. It was assumed that the value of a certain ratio in the appropriate percentile of its distribution reflects the percentage risk of rupture in the study group. The values of the ratios that had significant differences between groups were arbitrarily selected for the 20th, 25th and 30th percentiles as it was decided that the information about such a risk would be most clinically useful if the role of the ratios were to be more widely recognized. On the basis of the obtained results, it can be noticed that in most of the analyzed cases the ratios tended to be greater than or equal to 2 in the case of ruptured aneurysms, while in the case of non-ruptured aneurysms, the ratio exceeds this value only in sporadic cases. In other words, a 20% or greater risk of aneurysm rupture in this study group is when the aneurysm is at least twice the diameter of the unchanged aorta.

In our opinion, the obtained results constitute an interesting and helpful aspect for predicting the risk of AAA rupture and identifying patients particularly at risk of this incident. The role of the ratios calculated in this way should be verified in other research groups and taking into account additional risk factors. The use of additional techniques such as FEM for the assessment of PWS within individual AAA and RAAA could be particularly helpful in verifying the usefulness of the ratios [30]. For example, Urrutia et al. observed that PWS, calculated using FEM, apart from the maximum size of the aneurysm, is equally well correlated with other parameters such as: T (tortuosity), DDr (maximum diameter to neck diameter ratio), S (wall surface area), K-median (median of the Gaussian surface curvature), C-max (maximum lumen compactness), and M-mode (mode of the mean surface curvature) [31]. Zelaya et al. proposed estimating the PWS using a so-called linear model. The authors compared the vascular wall tension between patient-specific AAA models with the results obtained using conventional approaches and a hypothetical AAA reference model. It has been shown that such a linear model allows for a simple and effective estimation of PWS on the basis of a single CT scan, without referring to complicated analytical methods [32].

In recent years, there were some other attempts to assess the risk of AAA rupture based on aneurysm morphology. Netio-Palomo et al. were performing complex and detailed statistical analysis of individual aneurysm risk of rupture based on its size, shape and wall stress [33]. The authors made some very interesting observations linking the increased risk of aneurysm rupture with parameters other than the maximum diameter, such as the length of the aneurysm, its symmetry coefficient or the way blood flows through the aorta. The analysis is based on the creation of an accurate three-dimensional graphic model of a given aneurysm and it allows for much more precise measurements than in the case of the ratios calculated by us, but the difficulty of its implementation and the precise criteria that a given aneurysm had to meet in order to be analyzed determined the small size of the study group. For the same reason, ruptured aneurysms were not included in the study, so it is not possible to make any comparisons using the technique presented by the authors in this field.

An approach to the analyzed problem similar to ours was presented by Vande Geest et al. In their work, the authors calculated the rupture potential index (RPI), which is the ratio containing the relation of wall stress to wall strength and they compared it between patients with ruptured and non-ruptured AAA [33]. On the basis of the calculations performed, no differences were found between the groups, but their small number makes one ask whether the studied individuals were group-representative enough to abandon this direction of research.

In our work, we emphasized the simplicity of calculations, which, although limiting their accuracy, allowed us to create a larger group than in many of the cited studies [33,34,35]. We hoped that observing a given feature in a larger population would emphasize its significance and we believe that further development of the study group, especially with patients from other centers, would confirm the significance of the ratios we calculated. We realize that taking into account the individually variable dimensions of the aorta when estimating the risk of aneurysm rupture is only one of many elements of the individual characteristics of the patient that could be included in the calculations. Apart from increasing the size of the study group, the use of basic biometric data could be extremely valuable in this aspect. In our study, we showed that for two patients with the same size of AAA, the one with a smaller aorta has a higher risk of rupture, which confirms the significance of individual differences between patients. The most obvious physical difference between people is, of course, their height and weight. Incorporating these data in correlation with aortic and aneurysm sizes could significantly affect the reliability of the data, and this is a direction that should be followed in future analyses. Unfortunately, although these data appear to be easy to obtain, in some severe and unstable cases of RAAA, it is not possible to precisely measure height and weight or obtain them from the patient.

The results obtained by our team and other authors seems to be promising enough to consider the gathering of missing data and complexing the calculations with more advanced three-dimensional models. If that had been achieved, we would have had truly individual abdominal aortic aneurysm rupture risk assessment.

## 5. Conclusions

The ratio of aneurysm size to the size of the unchanged aorta visibly differs between ruptured and unruptured aneurysms, which results from the larger maximum size of ruptured aneurysms. In the study group, while maintaining the same size of the aneurysm, the aorta was smaller in ruptured aneurysms than in non-ruptured aneurysms. In the case of small aneurysms, their maximum size does not determine the risk of rupture high enough to predict this incident. The ratio of the aneurysm diameter to the iliac arteries is not applicable in assessing the risk of rupture. The ratio of the aneurysm sac to aorta diameter is a better prognostic factor for rupture, but only in small aneurysms with a present neck segment. In the study group, the aneurysm size enlarging twice as compared to the unchanged aorta determined at least a 20% risk of aneurysm rupture. In order to confirm the usefulness of calculated ratios for the assessment of the risk of rupture, further studies on other groups of patients are necessary.

## Data Availability

Data available on request from corresponding author.
